# Metastasis of aggressive amoeboid sarcoma cells is dependent on Rho/ROCK/MLC signaling

**DOI:** 10.1186/1478-811X-11-51

**Published:** 2013-07-30

**Authors:** Jan Kosla, Daniela Paňková, Jiří Plachý, Ondřej Tolde, Kristýna Bicanová, Michal Dvořák, Daniel Rösel, Jan Brábek

**Affiliations:** 1 Department of Cell Biology, Faculty of Science, Charles University in Prague, Viničná 7, 12843, Prague 2, Czech Republic; 2 Laboratory of Molecular Virology or Cellular and Viral Genetics, Institute of Molecular Genetics, Academy of Sciences of the Czech Republic, Vídeňská 1083, 14220, Prague 4, Czech Republic

**Keywords:** Metastasis, Sarcoma, RhoA, ROCK, MLC, Amoeboid invasiveness, 3D environment, Chicken model

## Abstract

**Background:**

Although there is extensive evidence for the amoeboid invasiveness of cancer cells in vitro, much less is known about the role of amoeboid invasiveness in metastasis and the importance of Rho/ROCK/MLC signaling in this process.

**Results:**

We analyzed the dependence of amoeboid invasiveness of rat and chicken sarcoma cells and the metastatic activity of chicken cells on individual elements of the Rho/ROCK/MLC pathway. In both animal models, inhibition of Rho, ROCK or MLC resulted in greatly decreased cell invasiveness in vitro, while inhibition of extracellular proteases using a broad spectrum inhibitor did not have a significant effect. The inhibition of both Rho activity and MLC phosphorylation by dominant negative mutants led to a decreased capability of chicken sarcoma cells to metastasize. Moreover, the overexpression of RhoA in non-metastatic chicken cells resulted in the rescue of both invasiveness and metastatic capability. Rho and ROCK, unlike MLC, appeared to be directly involved in the maintenance of the amoeboid phenotype, as their inhibition resulted in the amoeboid-mesenchymal transition in analyzed cell lines.

**Conclusion:**

Taken together, these results suggest that protease-independent invasion controlled by elements of the Rho/ROCK/MLC pathway can be frequently exploited by metastatic sarcoma cells.

## Background

Cancer metastasis is a multistage process composed of series of phenotypic and biochemical changes, including altered gene expression, angiogenesis, lymphangiogenesis, motility and cell shape [1]. During the first step of metastatic spreading, the malignant tumor cells initiate separation from the primary tumor mass and break contacts with neighboring cells. Then, the tumor cells degrade and penetrate the extracellular matrix and enter the bloodstream or lymphatic system, from where they can exit at a new site and proliferate in secondary organs [2]. The most critical steps in metastasis are the cell migration and cell invasion that are responsible for the malignancy of tumor cells invading the surrounding tissues [3-5]. This is represented by dynamic filamentous actin cytoskeletal remodeling, which enables tumor cells to adhere to the extracellular matrix and generate intracellular forces for cell movement [3-5]. Actin remodeling and the whole process of cell movement are regulated by small GTPases of the Rho family, mainly RhoA, RhoC, cdc42 and Rac [6,7]. Nevertheless the capacity of tumor cells to invade adjacent tissues depends on specific migratory mechanisms.

There are two main types of movements adopted by tumor cells, amoeboid and mesenchymal, and it has been shown that the Rho/ROCK and Rac signaling pathways are critical for both [8,9]. Mesenchymal movement requires integrin attachment to the extracellular matrix, the formation of strong focal contacts, and pericellular proteolysis. Cells migrating by the mesenchymal mode also display an elongated morphology in the three-dimensional (3D) environment [10]. In contrast, some tumor cells can move with an amoeboid, rounded shape that is associated with the formation of small membrane blebs and cortical actin [9]. In amoeboid tumor cells the activation of Rho and its downstream kinase ROCK leads to the increased generation of traction forces [11], allowing the amoeboid cells to push through the extracellular matrix independently of extracellular matrix degradation [11,12]. ROCK kinase is subsequently suggested to affect the traction forces by phosphorylation of the myosin light chain (MLC), which activates actomyosin contractility [13,14].

Although there is extensive evidence for the amoeboid invasiveness of cancer cells in vitro [15] and its dependence on Rho/ROCK/MLC signaling [9], much less is known about the plausibility of amoeboid invasiveness and metastasis in vivo and the importance of Rho/ROCK/MLC signaling in this process [12,16,17]. In this study we analyzed the role of the individual components of Rho/ROCK/MLC signaling for morphology, invasion and, importantly, also for the metastatic potential of amoeboid sarcoma cells.

## Results

### The Rho/ROCK/MLC pathway is critical for the invasion of highly metastatic rat A3 cells into the 3D collagen matrix and acellular dermis

**Figure 1:**
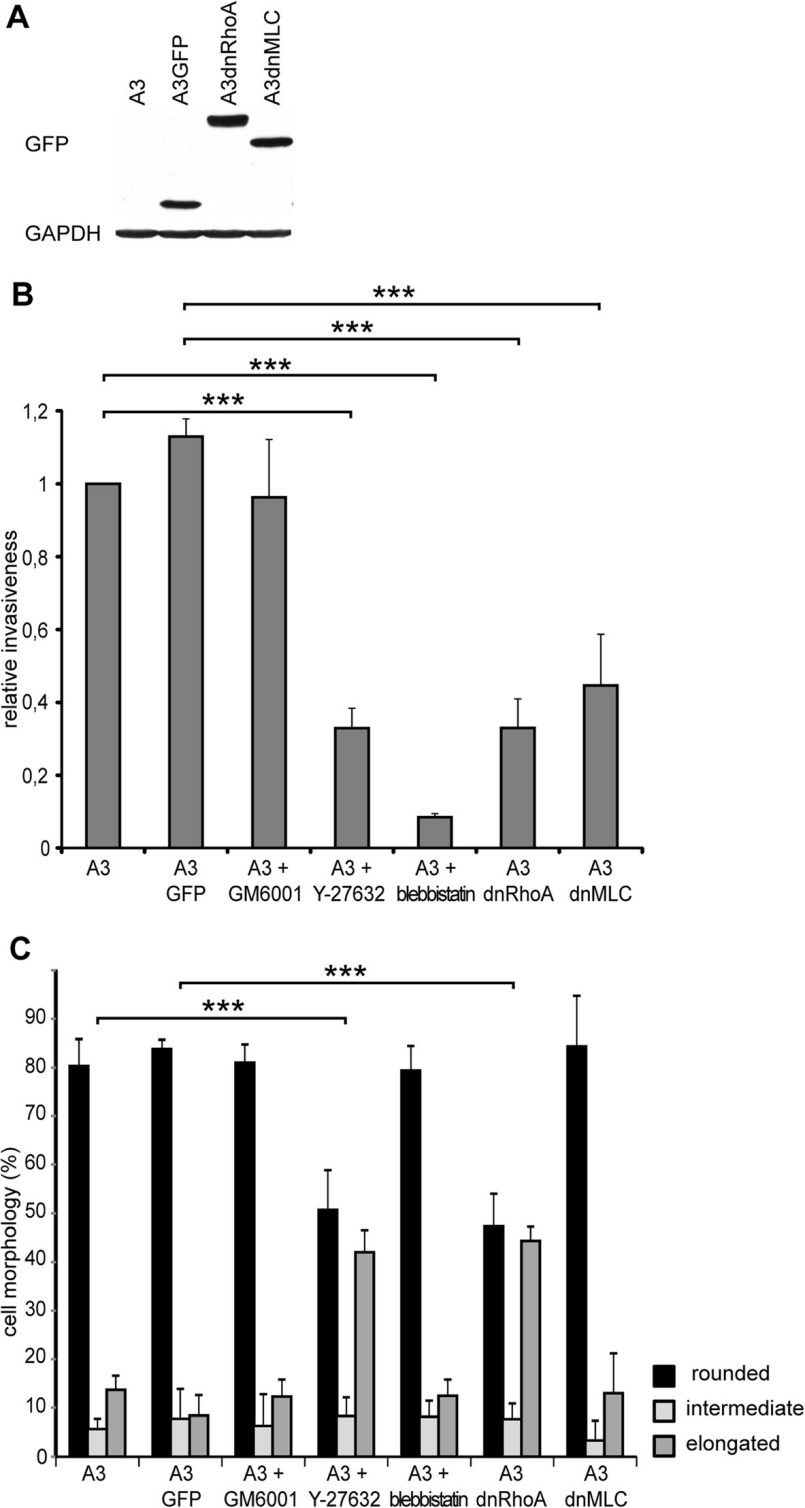
**Effects of Rho, ROCK, MLC signaling inhibition on the invasiveness and morphology of A3 rat cells. (A)** Immunodetection of GFP-tagged RhoA and MLC proteins in A3 cells. **(B)** The in vitro invasion of A3 cells in 3D collagen. Treatment of A3 cells with metalloproteinase inhibitor GM6001 has no significant effect on invasion. Inhibition of ROCK by Y-27632 and inhibition of non-muscle myosin II ATPases activity by Blebbistatin in A3 cells as well as inhibition of both RhoA in A3dnRhoA cells and MLC in A3dnMLC cells leads to decreased ability of these cell lines to invade 3D collagen. **(C)** Morphology of A3 cells in 3D collagen in vitro. Activity of RhoA and ROCK is required for rounded morphology of the A3 cell line. Inhibition of ROCK by Y-27632 and inhibition of RhoA in A3dnRhoA lead to the amoeboid-mesenchymal transition in 3D collagen. Inhibition of metalloproteinases by GM6001, inhibition of non-muscle myosin II ATPases activity by Blebbistatin in A3 cells and inhibition of MLC activity in A3dnMLC cells has no significant effect on morphology.

In our previous study [11], we showed for the first time that highly metastatic A3 rat sarcoma cells use the Rho/ROCK-dependent amoeboid mode of invasion as their primary invading mechanism. Furthermore, we showed that the up-regulation of Rho/ROCK signaling results in an increased invasion into Matrigel associated with the increased activation of Rho and recruitment of the phosphorylated myosin light chain (MLC) to the leading edge of the cell. To elucidate the role of individual components of Rho/ROCK/MLC signaling in the amoeboid invasiveness of sarcoma cells, cell lines were prepared to stably express either GFP-tagged dnRhoA (A3dnRho; inactivating mutation T19N) or GFP-tagged non-phosphorylable (dominant negative) MLC (A3dnMLC; mutations T18A, S19A) in A3 cells. Control cells (A3GFP) expressing only GFP were prepared as well. The presence of GFP-tagged recombinant proteins in A3 cells was confirmed by immunodetection (Figure 1A) and the resulting cell lines were analyzed for invasiveness and morphology in 3D collagen. Consistently with our previous analysis of the invasiveness of A3 cells by a Matrigel invasion assay [11], we found that A3 cell invasiveness in 3D collagen is greatly decreased in the presence of ROCK inhibitor Y-27632 or non-muscle myosin II ATPases activity inhibitor Blebbistatin and insensitive to the presence of a broad-spectrum metalloproteinase inhibitor, GM6001. Expression of both dominant negative Rho and MLC resulted in the greatly decreased capability of cells to invade a 3D collagen gel (Figure 1B), confirming their dominant negative effect on Rho and MLC signaling, respectively. Next, we analyzed the effect of Rho/ROCK/MLC inhibition on the morphology of cells in 3D collagen. Inhibition of Rho activity by the expression of dnRhoA or inhibition of ROCK by Y-27632 led to the amoeboid-mesenchymal transition in A3 cells. Surprisingly, inhibition of MLC activity by the expression of dnMLC and inhibition of non-muscle myosin II ATPases activity by Blebbistatin, despite their inhibitory effect on invasiveness, did not led to a significant change in A3 cell morphology in 3D collagen (Figure 1C and Additional file 1: Figure S1).

**Figure 2:**
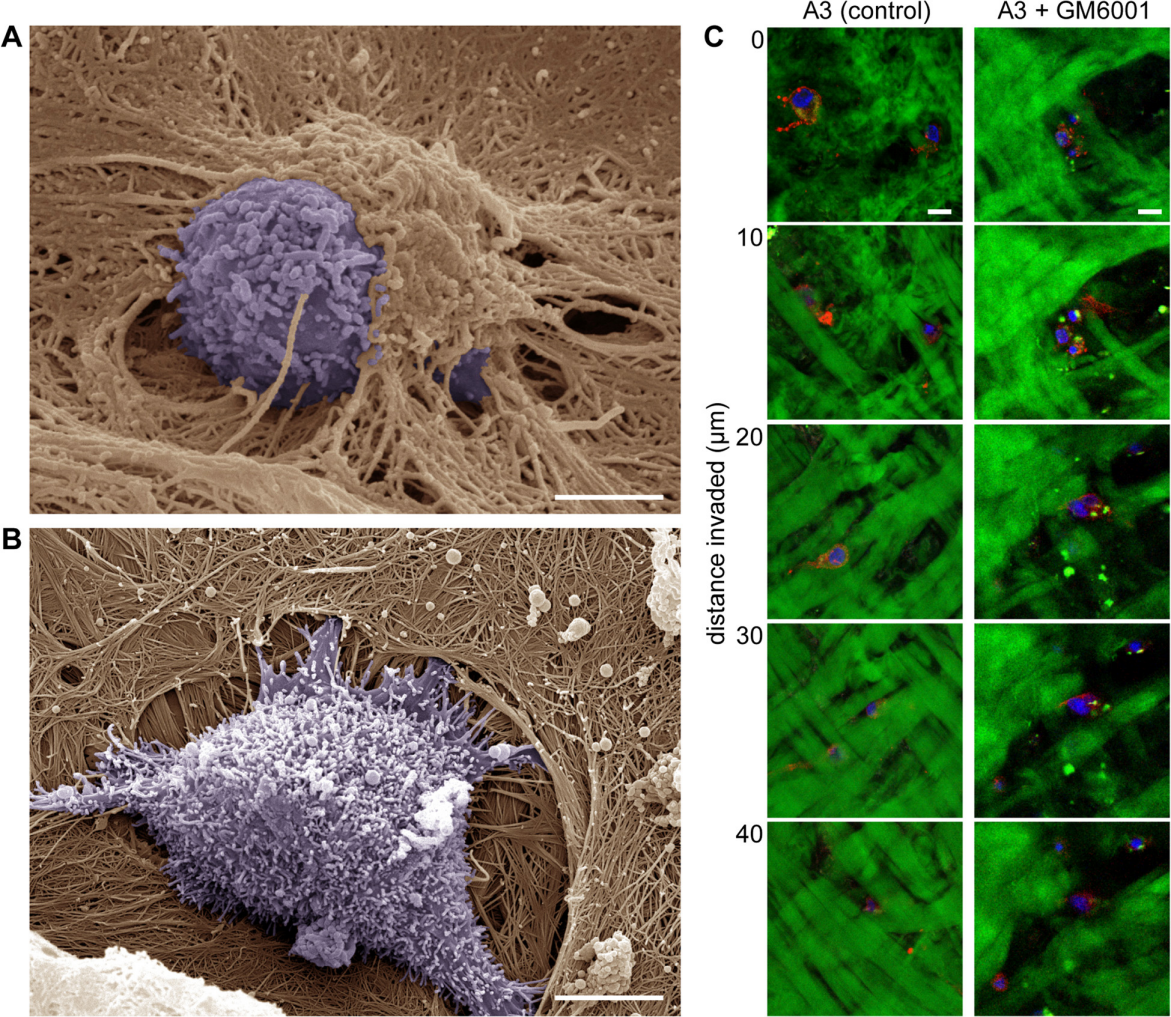
**A3 cells use the amoeboid mode of invasion.** Initial steps of dermis-based matrix invasion were visualized by scanning electron microscopy. **(A)** A3 cells maintain rounded morphology, and during invasion they appear to push themselves under extracellular matrix fibers. Scale bar 5 μm. **(B)** RsK4 cells are positioned partially inside a cavity and are surrounded with wrapped fibers. Scale bar 5 μm. **(C)** Invasive capability of A3 cells is not affected by the presence of 20 μM metalloproteinase inhibitor GM6001 (as also shown in Figure 1). A3 cells were seeded on FITC-stained dermis (green) and after 3 days were fixed and stained by Phalloidin for F-actin (red) and by DAPI for nuclei (blue). The images series represent serial optical sections captured at 10 μm intervals from top of dermis-based matrix (0 μm) to 40 μm of invasion depth. Scale bars 10 μm.

To visualize the shape of cells and interactions between cells and the extracellular matrix in detail, the cells were seeded on a dermis-based matrix and visualized by scanning electron microscopy (SEM). We have previously successfully used this substrate, which closely resembles the biochemical and biomechanical properties of the matrix in tissues, to elucidate the structure of invadopodia [18] as well as the structure and dynamics of focal adhesions [19] in a complex 3D environment. The A3 cells visualized by SEM during the initial steps of dermis-based matrix invasion exhibited a rounded morphology and did not seem to cleave collagen fibers. Rather, they appeared to push themselves beneath the sheet of collagen fibers (Figure 2A). This rounded morphology and invasion without obvious cleavage of collagen fibers was also observed for A3 cells invading isolated fascia of a rat diaphragm (Additional file 2: Figure S2). In contrast, closely related but mesenchymally invading RsK4 cells [18] were typically positioned partially inside a cavity in the dermis, which was apparently formed by the degradation of matrix fibers (Figure 2B). 3D scanning of the dermis-based matrix by confocal microscopy further revealed that A3 cells effectively invaded the matrix even in the presence of a broad-spectrum MMP-inhibitor, without noticeable degradation of the collagen fibers (Figure 2C). In contrast and consistent with the SEM results (Figure 2B), K4 cells have previously been shown to extensively degrade the matrix in the absence of inhibitor, whereas in the presence of an MMP inhibitor they were not able to invade and the matrix remained mostly intact [18].

### The Rho/ROCK/MLC pathway is critical for the invasion of highly metastatic chicken PR9692 sarcoma cells into a 3D collagen matrix

**Figure 3:**
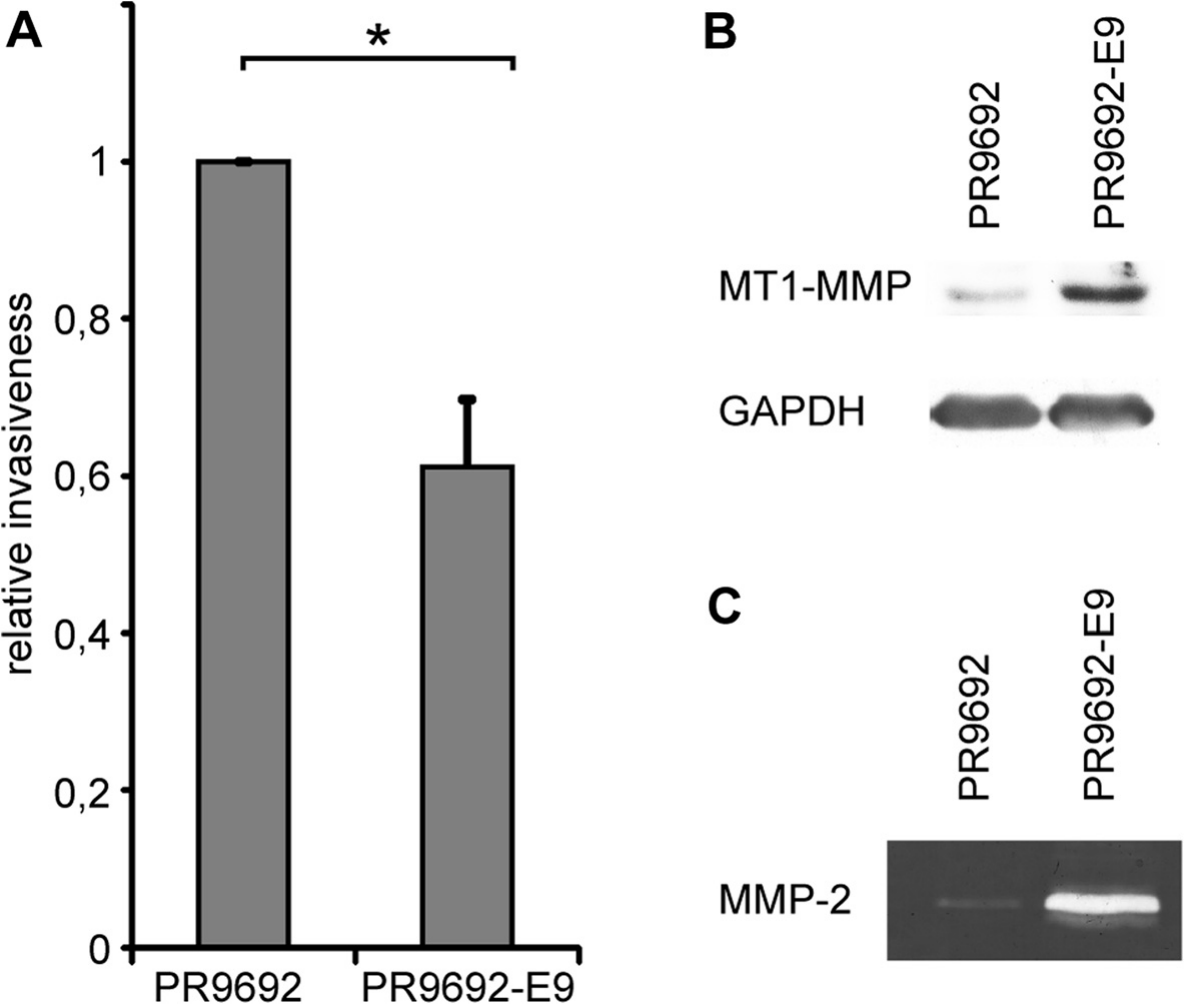
**Metastatic PR9692 cells adopted the amoeboid mode of invasion while non-metastatic PR9692-E9 cells use the mesenchymal mode. (A)** 3D in vitro collagen invasion. **(B)** Immunochemical detection of MT1-MMP (MMP14) protein levels. **(C)** Activity of MMP-2 metalloproteinase detected by gelatin zymography.

**Figure 4:**
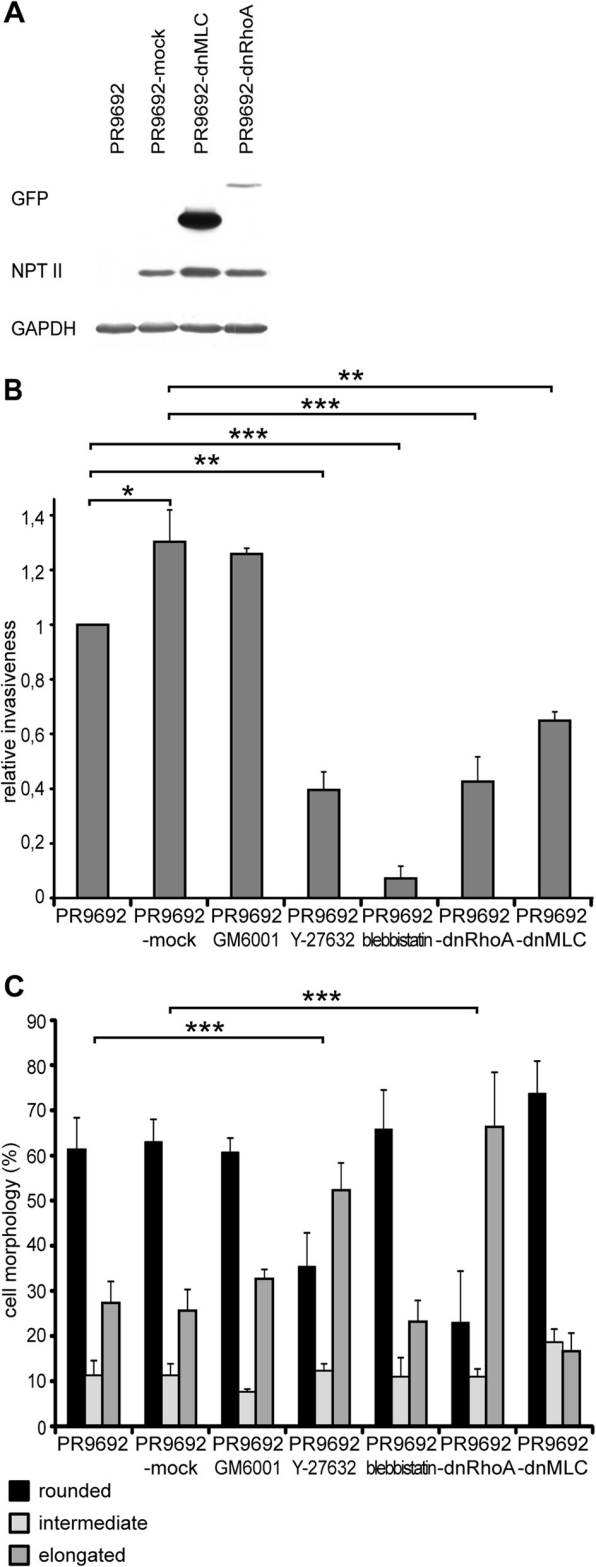
**Effect of Rho, ROCK, MLC signaling inhibition on the invasiveness and morphology of PR9692 cells. (A)** Immunodetection of recombinant dnRhoA, dnMLC and NPTII proteins in PR9692 cells. **(B)** 3D in vitro collagen invasion. Treatment of PR9692 cells with metalloproteinase inhibitor GM6001 does not reduce the cell invasion. Inhibition of ROCK by Y-27632 and inhibition of non-muscle myosin II ATPases activity by Blebbistatin in PR9692 cells as well as inhibition of both RhoA in PR9692-dnRhoA and MLC in PR9692-dnMLC lead to decreased ability of these cell lines to invade 3D collagen. **(C)** Morphology assay in 3D collagen in vitro. Activity of RhoA is required for the rounded morphology of the PR9692 cell line. Inhibition of ROCK by Y-27632 and inhibition of RhoA in PR9692-dnRhoA lead to the amoeboid-mesenchymal transition in 3D collagen. Inhibition of metalloproteinases by GM6001, inhibition of non-muscle myosin II ATPases activity by Blebbistatin in PR9692 cells and inhibition of MLC activity in PR9692-dnMLC cells have no significant effect on morphology.

To verify our observations of amoeboid invasiveness in rat sarcoma cells, we next wanted to analyze an independent amoeboid sarcoma cell line from another organism. We chose a chicken sarcoma model of metastasis consisting of the parental highly metastatic PR9692 cell line and the non-metastatic PR9692-E9 cell line (a subclone of PR9692 cells) [20]. Both PR9692 and PR9692-E9 cells give rise to rapidly growing sarcomas. While tumors induced by parental PR9692 cells efficiently metastasize into lungs, PR9692-E9 tumors never metastasize. Microarray analysis comparing metastatic PR9692 cells to non-metastatic PR9692-E9 have revealed an almost 80 fold increased expression of *myl9* (myosin regulatory light chain 2, *mlc*2) mRNA in PR9692 cells [20], suggestive of the potentially increased actomyosin contractility of PR9692 cells. Using the 3D invasion assay we confirmed that metastatic PR9692 cells are more invasive than non-metastatic PR9692-E9 cells (Figure 3A). An analysis of morphology in 3D collagen revealed that PR9692 cells adopt a rounded morphology in a 3D environment (Figure 4C, Additional file 1: Figure S1).

To confirm the amoeboid phenotype of PR9692 cells we tested their sensitivity to ROCK inhibitor as well as the expression of extracellular matrix proteases. The analyses revealed that PR9692 cells produce smaller amount of both MT1-MMP (MMP14) and MMP-2 than PR9692-E9 cells (Figure 3B and C). The addition of ROCK inhibitor to PR9692 cells greatly inhibited their invasiveness, even below the invasive capacity of PR9692-E9 (Figures 3A and 4B), and induced an effective amoeboid-mesenchymal transition (Figure 4C, Additional file 1: Figure S1). Conversely, the cells were insensitive to the broad-spectrum metalloproteinase inhibitor GM6001 (Figure 4C). Taken together, these results confirm the amoeboid nature of PR9692 cells. To inhibit RhoA and MLC signaling in PR9692 cells, replication-defective viruses encoding dominant negative RhoA (dnRho; inactivating mutation T19N) or non-phosphorylable MLC (dnMLC; mutations T18A, S19A) were used to infect PR9692 cells. The resulting cells were screened for the presence of GFP-tagged dnRhoA and dnMLC by immunoblotting. Detected protein levels of dnRhoA and dnMLC varied, probably reflecting the cellular regulation of these proteins’ different stability, as the extent of viral integration and expression in infected cells shown by the immunodetection of neomycin phosphotransferase II (NPT II) was very similar (Figure 4A).

We then explored the effect of Rho, MLC and non-muscle myosin II ATPases activity inhibition on PR9692 cell invasiveness in 3D collagen. We found that all Rho, MLC and non-muscle myosin II ATPases activity inhibition resulted in great decrease of the capability of PR9692 cells to invade a 3D collagen gel (Figure 4B). Next, we analyzed the effect of Rho/ROCK/MLC inhibition on the morphology of cells in 3D collagen. We found that while inhibition of Rho activity by the expression of dnRhoA or inhibition of ROCK by Y-27632 led to the amoeboid-mesenchymal transition, MLC inhibition, treatment with the metalloproteinase inhibitor GM6001 or non-muscle myosin II ATPases activity inhibitor Blebbistatin did not lead to a significant change in cell morphology in 3D collagen (Figure 4C, Additional file 1: Figure S1). Taken together, these results suggest the important role of RhoA and ROCK activity as well as the phosphorylation of MLC and non-muscle myosin II ATPases activity in the invasiveness of highly metastatic PR9692 sarcoma cells into 3D collagen.

### The Rho/ROCK/MLC pathway is critical for the metastatic capability of PR9692 cells

**Figure 5:**
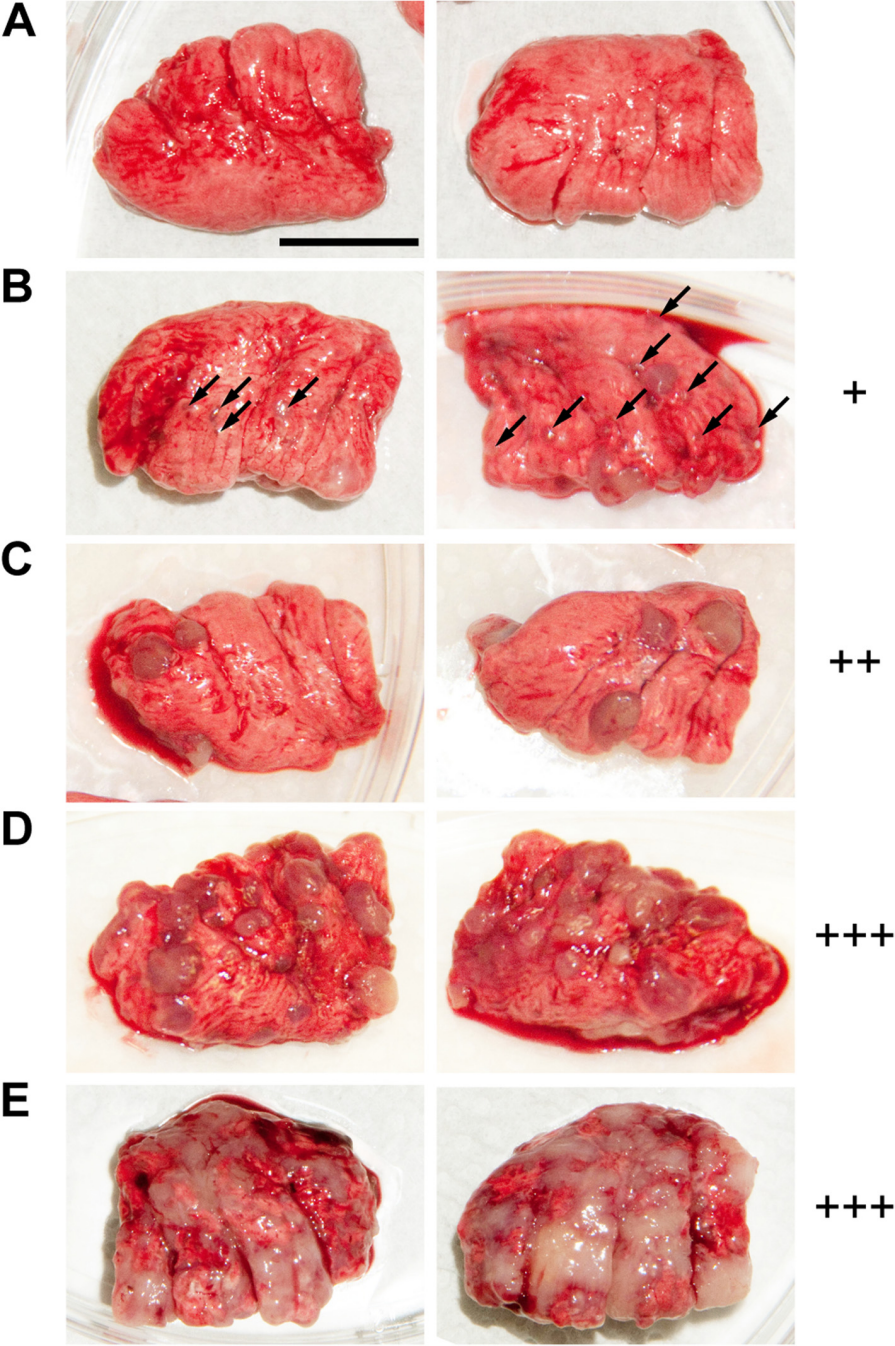
**Categories of lung metastases according to sizes and numbers of tumors. (A)** no metastases **(B)** +, multiple (>10) small (indicated by arrowheads) and 1–3 medium sized (3–4 mm) metastases; **(C)** ++, multiple small and more than 3 medium sized metastases and/or 1–3 large (4–5 mm) isolated metastases; **(D)** +++, lungs overgrown with medium to large metastases growing together, frequently more than 50% of lungs formed by tumor tissue; **(E)** in some chicks metastases seized the majority of the lungs. Scale bar 1 cm.

**Figure 6:**
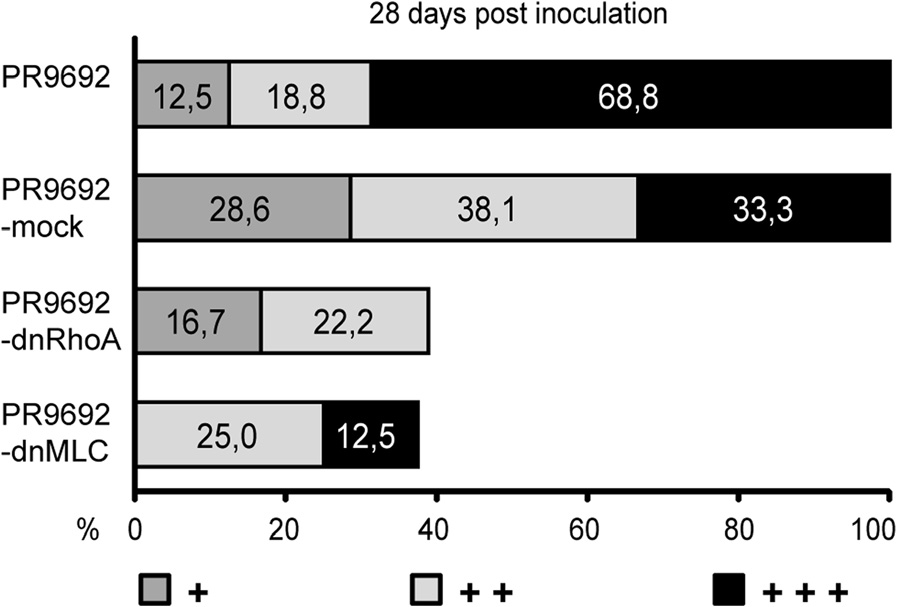
**Effect of Rho and MLC signaling inhibition on the metastatic activity of PR9692 cells.** Inhibition of RhoA signaling in PR9692-dnRhoA and inhibition of MLC activity in PR9692-dnMLC led to the decreased metastatic activity of PR9692 cells.

To examine the role of RhoA activation and MLC phosphorylation in the in vivo metastatic capacity of PR9692 cells, animals were injected with PR9692-mock, PR9692-dnMLC, and PR9692-dnRhoA cells. The animals were killed 21, 28 and 35 days post-injection (d.p.i.) and their lungs inspected for the presence of metastases. The extent of metastases was expressed by three categories representing the number and size of metastases. Examples of these three categories are shown in Figure 5. The size of the primary tumor did not correlate with the number and size of metastases or proliferation rate of cells in vitro as there is basically no effect of caRhoA, dnRhoA or dnMLC on growth of cells in comparison with MOCK in vitro (Additional file 3: Table S1 and Additional file 4: Table S2). The difference in proliferation between infected (MOCK, caRhoA, dnRhoA and dnMLC) and not infected cells is negligible (maximally 7% of not-infected cells’ doubling time) (Additional file 3: Table S1 and Additional file 4: Table S2). We found that the inhibition of RhoA signaling in PR9692-dnRhoA and inhibition of MLC activity in PR9692-dnMLC (both lead to decreased invasion in 3D collagen in vitro) led to a great decrease in the metastatic activity of these cells. A reduction of metastatic spreading was detected in most of animals injected with either PR9692-dnRhoA or PR9692-dnMLC cells when compared with PR9692-mock cells or PR9692 cells. While PR9692-mock cells or PR9692 cells formed metastases in 100% of cases, only about 50% of animals injected with both PR9692-dnRhoA and PR9692-dnMLC gave rise to metastases. Importantly, the size and number of metastatic foci in animals injected with PR9692-dnRhoA was reproducibly smaller in comparison to those induced by PR9692-mock cells or PR9692 (Figure 6 and Additional file 3: Table S1). Taken together, these results suggest the important role of RhoA and ROCK activity as well as the phosphorylation of MLC in the in vivo metastasis of PR9692 sarcoma cells.

### Activation of RhoA in non-metastatic PR9692-E9 cells results in rescue of the invasive and metastatic capability of these cells

**Figure 7:**
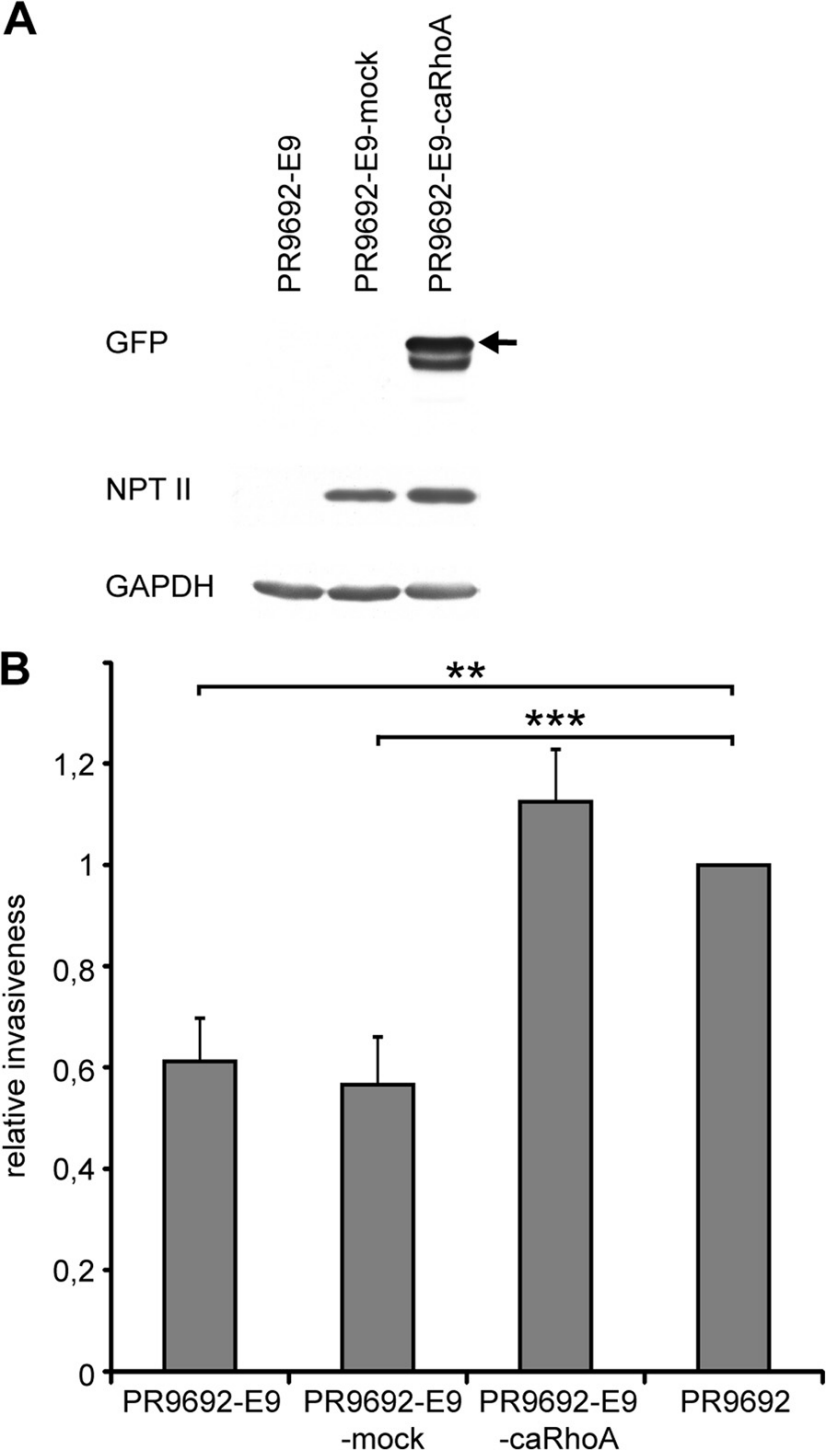
**Activation of RhoA leads to increased invasive potential in PR9692-E9 cells. (A)** Immunochemical analysis of recombinant caRhoA proteins in PR9692-E9-caRhoA cells. GFP-tagged full-length proteins are indicated by the arrowhead. **(B)** 3D in vitro collagen invasion.

**Figure 8:**
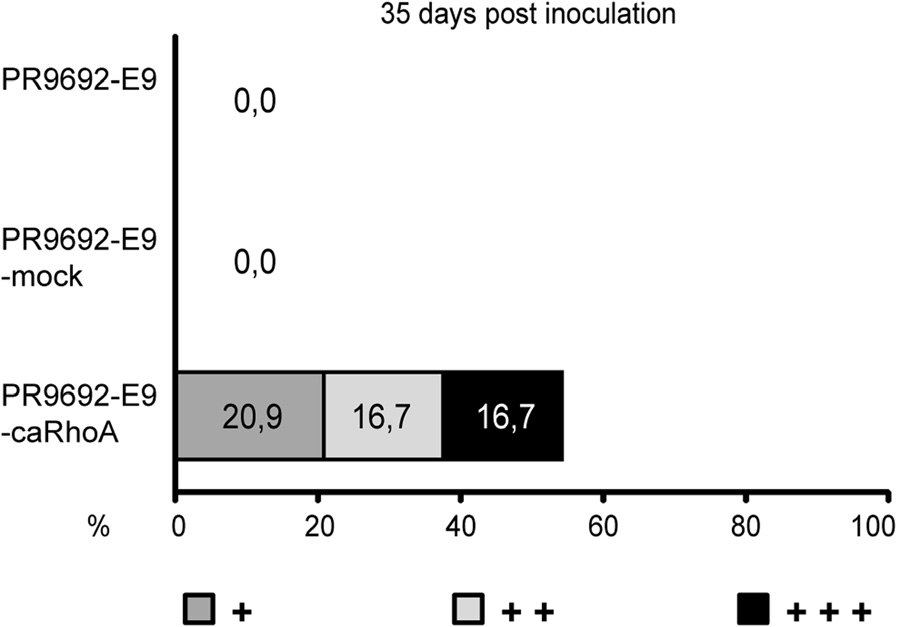
Activation of RhoA signaling in PR9692-E9-caRhoA cells led to the restoration of metastatic potential of PR9692-E9.

Finally, we wondered whether the activation of Rho/ROCK/MLC signaling through the expression of constitutive-active RhoA (caRhoA, activating mutationG14V) would result in rescue of the metastatic capability of non-metastatic PR9692-E9 cells. Replication-defective viruses encoding caRhoA were used to infect PR9692-E9 cells. Control cells infected with empty SFCV-LE retroviral vector (PR9692-E9-mock) were prepared in the same fashion. The resulting cells, resistant to G418, were screened for the presence of GFP-tagged caRhoA as well as NPT II by immunoblotting to show that the extent of viral integration and expression was very similar in both infected cells (Figure 7A). We first compared the invasive capability of PR9692-E9 and PR9692-E9-caRhoA cells in a 3D collagen invasion assay. We found that PR9692-E9-caRhoA cells have a much greater capability to invade 3D collagen compared to the PR9692-E9 cell line or PR9692-E9-mock cells (Figure 7B). Next we compared the metastatic potential of PR9692-E9, PR9692-E9-mock and PR9692-E9-caRhoA cells. Lungs of animals injected with PR9692-E9, PR9692-E9-mock or PR9692-E9-caRhoA cells were inspected for the presence of metastases 28, 35 and 45 days post-injection. We found that the activation of RhoA signaling in PR9692-E9-caRhoA cells led to a restoration of metastatic potential. The formation of metastases, including all three metastatic categories, was detected in about 55% of animals injected with PR9692-E9-caRhoA cells, while no metastasis was detected in animals injected with PR9692-E9 or PR9692-E9-mock cells (Figure 8 and Additional file 4: Table S2). Taken together, these results suggest that the activation of Rho/ROCK/MLC signaling through the expression of constitutive-active RhoA is sufficient to rescue the invasive and metastatic capability of non-metastatic PR9692-E9 cells.

## Discussion

Amoeboid invasiveness in vitro was for the first time described in 2003 [8,9] and has been studied extensively since then. However, its relevancy for invasiveness and metastasis in vivo is still not clear. Sabeh et al. [21] suggested that the amoeboid invasiveness of tumor cells observed in vivo can only occur under specific conditions and may not be an effective and widespread alternative to protease-dependent tumor cell migration [21]. Yet, several studies have provided evidence for the plausibility of amoeboid invasion in vivo and Rho/ROCK dependent metastasis.

Initially, indirect supportive evidence for possible involvement of amoeboid invasiveness in the process of in vivo metastasis came from clinical studies with ROCK kinase inhibitors (reviewed in [22]). The ROCK kinase inhibitor fasudil was shown to reduce the dissemination of cancer in the peritoneal cavity, blood-borne metastasis to the lung, and prevent the establishment of breast tumors in the mammary fat pad [23]. Similarly, Y-27632 was shown to inhibit the tumor growth and intrahepatic metastasis of hepatocellular carcinoma [24]. In none of these studies, however, were cells with a defined protease-independent amoeboid invasiveness used. At least two independent studies have associated signaling changes leading to microtubule destabilization with the rounded morphology associated with increased invasion and metastatic potential. Hager et al. observed that DIAPH3 silencing in human carcinoma cells resulted in microtubule destabilization, a rounded morphology, and enhanced MYPT1 phosphorylation associated with increased invasive capability and metastatic potential in mice [25]. Belleti et al. found that stathmin stimulated cell motility through the extracellular matrix in vitro and increased the metastatic potential of sarcoma cells in vivo. Accordingly, a less phosphorylable stathmin point mutant impaired ECM-induced microtubule stabilization and conferred a higher invasive potential, inducing a rounded cell shape coupled with amoeboid-like motility in three-dimensional matrices [26]. In neither of these studies, however, did the authors show that the invasion of the cells studied was protease-independent or Rho/ROCK dependent.

The most convincing evidence to date for the role of protease-independent invasiveness in the process of in vivo metastasis was given by the Chiarugi lab in a series of studies analyzing the role of EphA2 in the metastasis of melanoma [27] and prostate carcinoma [28] cells. The authors showed that EphA2 re-expression in B16 murine melanoma cells converts their migration style from a mesenchymal to an amoeboid-like nonproteolytic invasive program, giving rise to successful lung and peritoneal lymph node metastases. [27]. They also showed that EphA2 expression in prostate carcinoma cells results in Rho-mediated cell rounding and independence from metalloproteinases, associated with increased metastatic potential [28]. In the only study so far analyzing the influence of direct manipulation of Rho/ROCK signaling on invasive and metastatic potential, Belgiovine et al. investigated the acquisition and molecular regulation of the invasive capacity of neoplastically transformed human fibroblasts, which were able to induce metastatic sarcomas when injected into immunocompromised mice. The cells showed a rounded morphology in the 3D environment and their invasiveness was sensitive to ROCK inhibitor, but not to a matrix protease inhibitor. The increased invasiveness of the cells was associated with the reduced expression of RhoE, a cellular inhibitor of ROCK. The ectopic RhoE expression reduced their invasive ability in vitro and their metastatic potential in vivo [29].

In our study we used two well characterized and defined independent lines of primarily amoeboid metastatic sarcoma cells with no detectable gelatinase activity from two different organisms. The protease-independent and ROCK-dependent movement of both A3 and PR9692 amoeboid sarcoma cells was initially shown in a 3D collagen invasion assays. Furthermore, to analyze the amoeboid invasiveness in a complex 3D environment, we used our model of acellular dermis, a substrate with biochemical and biomechanical properties very similar to that in tissue that we previously successfully used to elucidate the structure of invadopodia [18]. Our highly metastatic A3 sarcoma cells were able to effectively invade the acellular dermis irrespective of the presence of MMP inhibitor, further confirming the capability of sarcoma cells to effectively invade in a tissue-like environment.

**Figure 9:**
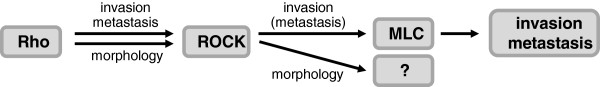
**Rho signaling in the maintenance of amoeboid morphology and invasion/metastasis of sarcoma cells.** The activation of Rho, ROCK and the phosphorylation of MLC are all crucial for the effective invasiveness/metastasis of amoeboid sarcoma cells. In contrast, the rounded morphology of amoeboid sarcoma cells is not dependent on MLC phosphorylation or non-muscle myosin II ATPase activity, suggesting another downstream effectors of Rho/ROCK pathway are involved in the maintenance of rounded morphology of cancer cells.

We show that the metastatic capability of amoeboid sarcoma cells in vivo is dependent not only on RhoA activity, but also on MLC phosphorylation status. We believe this is the first direct evidence for the importance of MLC phosphorylation status for the metastatic capability of cancer cells. Our data show that the activation of Rho, ROCK, the phosphorylation of MLC and non-muscle myosin II ATPases activity is necessary for effective invasiveness of both A3 and PR9692 sarcoma cells, and that the activation of Rho and MLC phosphorylation is necessary for the effective metastasis of PR9692 cells. However, our data also suggest that activation of Rho and ROCK but not MLC phosphorylation or non-muscle myosin II ATPases activity is required to maintain the rounded morphology of the sarcoma cells. This would imply a model (Figure 9) according to which signaling for the maintenance of rounded morphology is at least in part different from signaling enabling effective amoeboid invasiveness.

## Conclusions

Our study is the first study to show the effective amoeboid invasion and metastasis of cancer cells in a non-mammalian system, which further supports the general importance of the phenomena of amoeboid invasion and also opens up possibilities for introducing novel and powerful models, such as a syngeneic chicken model for the in vivo analysis of amoeboid cancer cell metastasis. Together with previous studies from the Mondello and Chiarugi labs, we believe our data provide the strongest evidence to date for the capability of amoeboid cancer cells to invade the tissue environment and effectively metastasize in vivo.

## Methods

### Establishment of stable cell lines and cell cultures

A3 cells (full name A337/311RP) and RsK4 cells were developed as described previously [30,31]. The A3dnRhoA and A3dnMLC cell lines were prepared to stably express either a GFP-fused dominant negative RhoA (mutations F25N, T19N; [32]) from pEGFP-dnRhoA or a non-phosphorylable (dominant negative) GFP-fused MLC (mutations T18A, S19A; [33]) by selection in G418 at 400 μg/ml and subsequent FACS sorting of GFP-positive cells (FACSVantage SE, BD Biosciences). Cells A3GFP were prepared in the same fashion using only empty pEGFP vector. Rat sarcoma cells were cultivated in full DMEM medium: DMEM (GIBCO) with 4500 mg/l L-glucose, L-glutamine, and pyruvate, supplemented with 10% fetal bovine serum (Sigma), 2% antibiotic-antimycotic (GIBCO) and 1% MEM non-essential amino acids (GIBCO) kept at 37°C in a humidified atmosphere with 5% CO_2_. The cell lines PR9692, PR9692-E9 and PR9692-E9-MOCK were established as described previously [20]. The cell lines PR9692-dnRhoA, PR9692-dnMLCMLC, PR9692-MOCK, and PR9692-E9-caRhoA were prepared in the same fashion as PR9692-E9-MOCK using the appropriate SFCV-LE vectors (SFCV-GFP-dnRhoA, SFCV-dnMLC-GFP, SFCV-LE, SFCV-GFP-caRhoA, respectively), the PR9692 or PR9692-E9 cell lines, and the KUNDRA packaging cell line. After transfection, individual clones of G418 resistant cells were tested for the expression of appropriate constructs by immunoblotting. All chicken cells were maintained in Dulbecco‘s modified Eagle’s medium (DMEM, Sigma) supplemented with L-glutamine, penicillin, streptomycin, 4% fetal calf serum (PAA) and 2% chicken serum (Sigma) at 41°C in a humidified atmosphere with 5% CO2. Cells were treated with inhibitors 20 μM GM6001 (Sigma), 10 μM Y-27632 (Sigma), and 50 μM Blebbistatin (Sigma). The doubling time of cell lines was determined as a mean value of three doubling times counted in consecutive passages of cells in exponential phase of growth.

### DNA constructs

SFCV-GFP-dnRhoA was constructed by introducing GFP-dnRhoA (EcoRI – NheI) from pEGFP-dnRhoA [32] between the XbaI and EcoRI sites of the pSFCV-LE vector. SFCV-GFP-caRhoA was constructed based on pEGFP-caRhoA (bearing RhoA with mutations F25N, G14V) [32] similarly to SFCV-GFP-dnRhoA. SFCV-dnMLC-GFP was constructed by introducing dnMLC-GFP (HindIII – NotI, Pfu polymerase blunted) from pEGFP-dnMLC [33] between the HindIII and EcoRV sites of the pSFCV-LE vector.

### Immunoblotting

For protein analysis, cells were plated onto 100-mm dishes and grown until subconfluent. Before lysis, the cells were transferred into 15-ml tubes and centrifuged. The pellets were dissolved in 500 μl of LysBuf (20 mM Tris pH 6.8, 1% Triton X-100, 0.3% SDS, 5 mM EDTA, 10% glycerol, protease inhibitor cocktail Complete, EDTA-free from Roche) and the lysate was aspirated and transferred to a 1.5 ml tube. The lysates were cleared by centrifugation at 15,000 rpm for 20 min. Protein concentration was assayed by the bicinchonic acid method (Pierce), the lysates were diluted to equal concentration with LysBuf, mixed with 5× SB (300 mM Tris pH 6.8, 5% SDS, 360 mM 2-mercaptoethanol, 50% glycerol, 0.05% bromphenol blue) and incubated at 99°C for 10 min. Proteins (40 μg/lane) were separated on a 10% polyacrylamide gel by SDS-PAGE and transferred to nitrocellulose membranes (Amersham Hybond-ECL). Membranes were probed with primary antibodies specific for GFP (sc-9996, Santa Cruz Biotechnology, 1:1,000), NPTII (#06-747, Millipore, 1:2,000) and MT-MMP1 (sc-12366, Santa Cruz Biotechnology, 1:1,000). Membranes were then incubated with the appropriate secondary antibodies (Jackson ImmunoResearch Laboratories) and subjected to enhanced chemiluminescence detection. For sequential detections, membranes were stripped with Re-Blot Plus Mild Antibody Stripping Solution (Millipore). Equal protein loading and transfer was verified by Ponceau-S staining of each membrane and by performing detection of GAPDH using antibody GTX30666 (GeneTex, 1:2,000) on the same membrane.

### In-gel gelatin zymography

In total, 2 × 10^5^ cells were plated per well in a 24-well plate. After 16 h, cells were washed with PBS and incubated in 300 μl of serum-free medium for 72 h. Aliquots (25 μl) of the conditioned medium were loaded for zymography on a 10% SDS-PAGE gel containing 1 mg/ml gelatin. Briefly, gel proteins were washed for 1 h in 50 mmol/l Tris–HCl (pH 7.5), 0.1 mol/l NaCl, and 2.5% Triton X-100 and then incubated at 37°C in 50 mmol/l Tris–HCl (pH 7.5), 10 mmol/l CaCl_2_, and 0.02% sodium azide for 17 h. The gels were stained with Coomassie blue and destained in 7% acetic acid/5% methanol.

### In vitro cell invasion assays in 3D collagen

The 3D collagen invasion assay was analyzed as described previously [34]. Briefly, cell suspension (2 × 10^5^ cells/ml) was added on top of a collagen gel in a multiwell plate, and after 48 hours the level of invasion was measured as the average invasion depth of the cells in the selected field of view using a Nikon-Eclipse TE2000-S (20×/0.40 HMC objective) and NIS-Elements software. For each experiment, invasion was analyzed in 3 wells and in 6 fields of view per individual well. In order to compare individual experiments, the average invasion depth was normalized to that of untreated cells. Three independent experiments were analyzed for each condition. Significance of differences was analyzed with ANOVA followed by Tukey’s honest significant difference test. The analysis was performed in version 2.15.3R (R Core Team, 2013. R: A language and environment for statistical computing. R Foundation for Statistical Computing, Vienna, Austria. ISBN 3-900051-07-0, URL http://www.R-project.org/).

### Cell morphology assays in 3D collagen

To analyze cell morphology in 3D collagen, cells were trypsinized, washed in complete medium, counted, and then 10^5^ cells were mixed with 500 μl of 3 mg/ml Collagen R in complete medium. This suspension of cells in collagen (500 μl) was loaded into a well of a 12-well plate, the gel was allowed to polymerize at 37°C for 30 min, and was then overlaid with complete medium. After 24 h the morphology of cells in 3D collagen was analyzed using the Nikon Eclipse TE2000-S microscope (20×/0,40 HMC objective). Cell morphology was classified on the basis of the elongation index. The elongation index was calculated as the length divided by the width. Cells whose elongation index was greater than 3 were considered elongated. Intermediate cells had an elongation index of 2–3; for rounded cells, the index was 1–2. Dividing cells were excluded from the analysis. Three independent experiments (at least 300 cells per experiment) were analyzed for each condition. As the data have the form of counts in categories, the Pearson's Chi-squared test was used to reveal statistically significant differences.

### Dermis-based matrix

For immunofluorescence staining the following procedure was used: two days before use the dermis-based matrix (XeDerma®; BIO SKIN a.s.) was cut into small pieces (approx. 1x1 cm), placed into 12 wells plates with HBSS buffer, and just prior to use washed twice with DMEM medium and cells were seeded on the epidermal side of the dermis. After incubation the dermis was washed with PBS and fixed. For labeling the dermis was pre incubated for two days in HBSS buffer. The conjugation of FITC (1 μg/ml; Molecular Probes) was performed in 0.1 M sodium bicarbonate buffer, pH.9.0 for 30 min and then the unconjugated dye was washed off three times with PBS and two times with DMEM.

### Scanning electron microscopy

Cells in full DMEM medium were grown on dermis-based matrix for 24 hours, and for the next 24 hours in serum free DMEM. The dermis-based matrix with cells was washed two times in PBS, fixed in 2.5% glutaraldehyde, and washed again three times. Dehydration in increasing concentrations of ethanol (10 min. each for 30%, 50%, 70%, 80%, 90%, 95 and 100%) was followed by critical point drying using CPD 030 (BAL TEC), coated with 3 nm gold on Sputter Coater SCD 050 (BAL TEC) and visualized by SEM on a JEOL 6380 LV. Rat fascia freshly removed from an adult rat was immobilized in a frame, positioned inside 6-well plates, and cells were seeded on top. After 2 days the whole frame was fixed and processed the same ways as dermis-based matrix.

### Immunofluorescence microscopy

Cells on epidermal side of dermis-based matrix were fixed in 4% paraformaldehyde, permeabilized in 0.5% Triton X 100, washed thrice with PBS, and stained for 15 min with Alexa 594 phalloidin (Molecular Probes) and then washed thrice with PBS and mounted in Mowiol containing 4',6-diamidino-2-phenylindole (DAPI, Sigma). Images were acquired by a Leica TCS SP2 microscope system using a Leica 20×/0.7 oil objective.

### Animals

Experiments were done with the Prague inbred chicken line CC.R1 [35]. All procedures were performed in accordance with the Guide for the Care and Use of Laboratory Animals and approved by the Animal Care and Use Committee of the Academy of Sciences of the Czech Republic. Chicks were kept under standard laboratory conditions with free access to food and water.

### Monitoring of tumor weight and metastases

Chickens were inoculated by injection into the outer area of the pectoral muscle at an age of 3 weeks with 5×10^5^tumor cells that had been freshly harvested from the tissue culture and resuspended in 0.2 ml of cultivation medium. The weight of the primary tumor and spontaneous metastatic activity of each tumor cell line were determined by examining chickens autopsied from 21 to 35 and from 28 to 45 days after inoculation with cells derived from the PR9692 cells and PR9692-E9 cells, respectively. The time of autopsy reflected the health status of the animals. Metastases were observed by gross inspection and using a dissection microscope. The experiments were performed several times with a total number of at least 33 animals in each group, except for the control groups where the numbers were slightly reduced (26) to spare animals.

## Abbreviations

ROCK: Rho-associated protein kinase; MLC: Myosin light chain; 3D: Three-dimensional; SEM: Scanning electron microscopy.

## Competing interests

The authors declare that they have not competing interests.

## Authors’ contributions

JB, JK, DR contributed to the design of the study. JK, DP, JP, OT performed the experiments and analysis of data. JK and KB prepared cell lines. JB, JK, DP, MD, DR contributed to the writing of the manuscript. All authors read and approved the final version of this manuscript.

## Supplementary Material

Additional file 1: Figure S1 Effect of Rho, ROCK, MLC signaling inhibition on morphology of A3 and PR9692 amoeboid cells. Representative figures of cell morphology in 3D collagen in vitro; scale bars 100 μm. Click here for file

Additional file 2: Figure S2 Amoeboid mode of invasion of A3 tested on abdominal rat fascia. A3 cells maintain rounded morphology. No disrupted fibers, an indication of degradation activity, can be seen in proximity of the cells. Two representative images are shown. Scale bars 5 μm. Click here for file

Additional file 3: Table S1 Detailed description of in vivo experiments with cells derived from PR9692 cells. Detailed results including doubling time of cells, weight of primary tumor and metastatic category of appropriate lung metastases in particular animals. Click here for file

Additional file 4: Table S2 Detailed description of in vivo experiments with cells derived from PR9692-E9 cells. Detailed results including doubling time of cells, weight of primary tumor and metastatic category of appropriate lung metastases in particular animals. Click here for file
