# Supplementary figures and images for: Metastasis of aggressive amoeboid sarcoma cells is dependent on Rho/ROCK/MLC signaling
Source: Cell Commun Signal. 2013 Jul 30;11:51. doi: 10.1186/1478-811X-11-51 (PMC3735423; doi:10.1186/1478-811X-11-51)

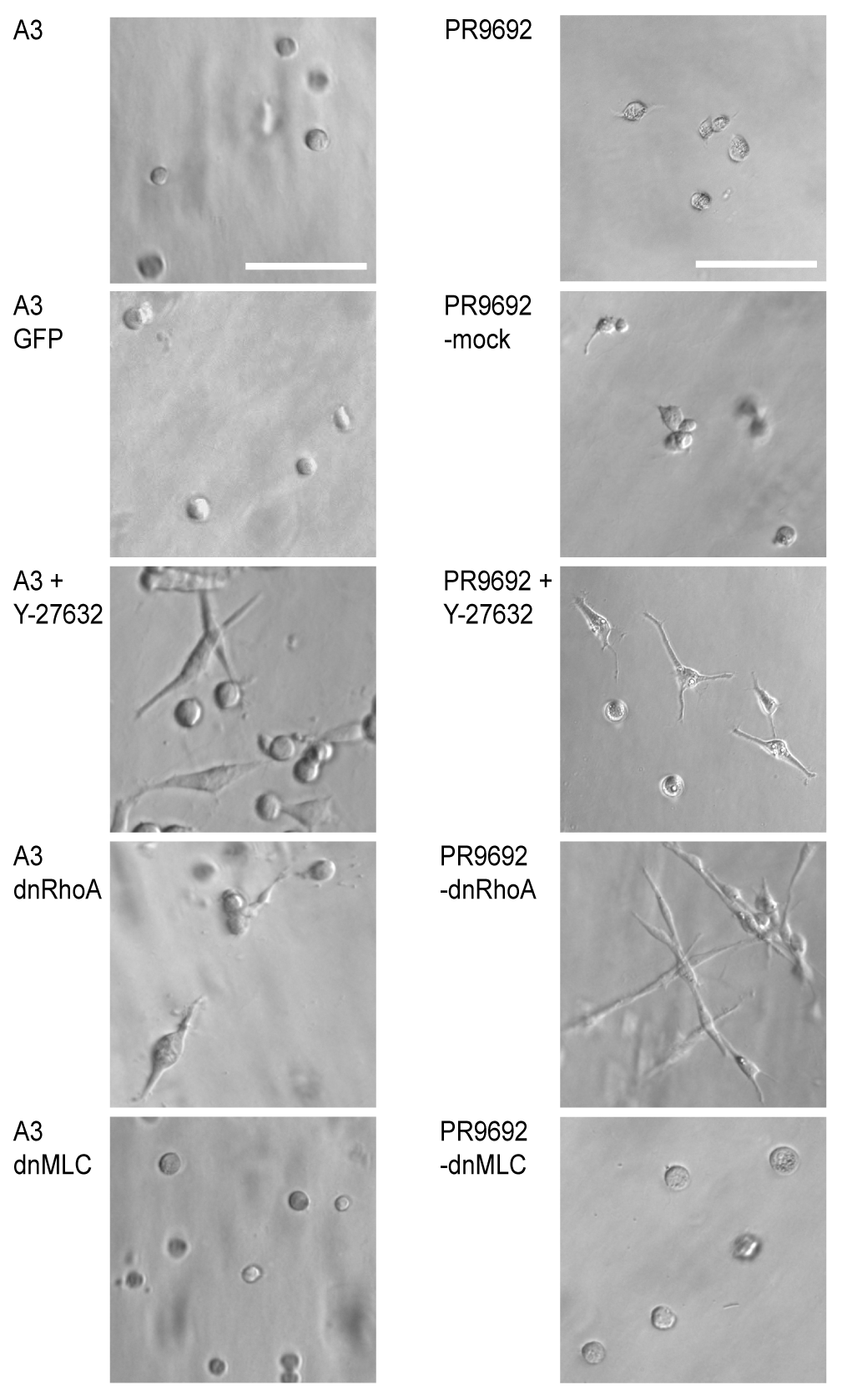

Supplement: Additional file 1: Figure S1 — Effect of Rho, ROCK, MLC signaling inhibition on morphology of A3 and PR9692 amoeboid cells. Representative figures of cell morphology in 3D collagen in vitro; scale bars 100 μm. [file 1478-811X-11-51-S1.tiff]

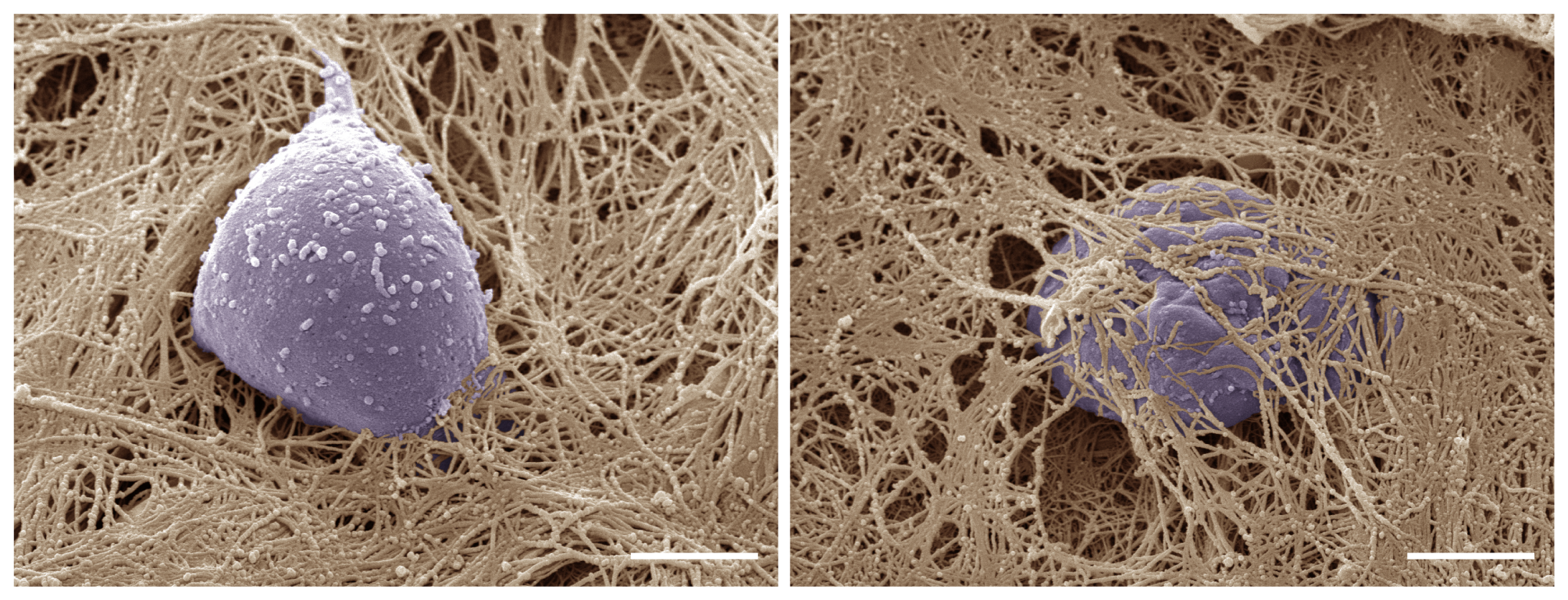

Supplement: Additional file 2: Figure S2 — Amoeboid mode of invasion of A3 tested on abdominal rat fascia. A3 cells maintain rounded morphology. No disrupted fibers, an indication of degradation activity, can be seen in proximity of the cells. Two representative images are shown. Scale bars 5 μm. [file 1478-811X-11-51-S2.tiff]
